# Three-Dimensional Finite Element Analysis of Different Connector Designs for All-Ceramic Implant-Supported Fixed Dental Prostheses

**DOI:** 10.3390/ceramics5010004

**Published:** 2022-01-05

**Authors:** Laura H. J. Alberto, Lohitha Kalluri, Josephine F. Esquivel-Upshaw, Yuanyuan Duan

**Affiliations:** 1 Department of Biomedical Materials Science, University of Mississippi Medical Center, Jackson, MS 39216, USA; 2 Department of Restorative Dental Sciences, Division of Prosthodontics, University of Florida, Gainesville, FL 32611, USA

**Keywords:** all-ceramic FPD, finite element analysis, gingival embrasure, connector design, dental implants

## Abstract

All-ceramic fixed dental prostheses (FDPs) tend to fracture at the connector regions due to high stress concentration at these areas influenced by their design. This study was performed as an adjunct to an existing clinical study to evaluate the influence of the different radii of curvature of gingival embrasure on the stress distribution of a three-unit all-ceramic implanted supported FDP. Three three-dimensional (3D) models were created by scanning two titanium dental implants, their suitable zirconia abutments, and a patient-retrieved dental prosthesis using a micro-CT scanner. The radius of curvature of the gingival embrasure for the distal connector of the FDP was altered to measure 0.25 mm, 0.50 mm, and 0.75 mm. A finite element analysis (FEA) software (ABAQUS) was used to evaluate the impact of different connector designs on the distribution of stresses. Maximum Principal Stress data was collected from the individual components (veneer, framework, and abutments). The radius of curvature of gingival embrasure had a significant influence on the stress distribution at the assessed components. The tensile peak stresses at all structures were highest in the 0.25 mm model, while the 0.50 mm and 0.75 mm models presented similar values and more uniform stress distribution.

## 1. Introduction

The development of ceramics with better physical properties [1-4], along with the inherent characteristics of this material (biocompatibility and superior aesthetics) [4], and the public demand for metal-free prosthesis [5-9] have made all-ceramic FDP gain popularity and acceptance in the dental community over metal-ceramics. Among the ceramic materials, yttria-stabilized tetragonal zirconia polycrystal (3Y-TZP) demonstrates high flexural strength (800–1500 MPa) [10] and fracture resistance, which makes 3Y-TZP the first choice for framework structure in all-ceramic dental prosthesis systems, especially in posterior sites [1,11,12], with survival rates of 90.4% after 5 years [13] and 91.3% after 10 years [14]. Despite their excellent mechanical characteristics, zirconia ceramics are usually opaque and need a veneer layer, usually glass-ceramic, to mimic the natural aesthetics of teeth [8,15-17].

Dental implants have become the treatment of choice for replacing missing teeth in partial or totally edentulous areas [18], even in patients with systemic conditions [19]. A meta-analysis demonstrated that implant-supported zirconia-ceramic FDPs have estimated survival rates of 93% for 5 years [20]. An all-ceramic implant-supported FDP under masticatory forces behaves like a ceramic beam on rigid supports. In this clinical scenario, compression is applied on the occlusal surface of the pontic and retainers, and tension is developed in the gingival aspect. This set of forces plays an important role in all-ceramic structures [10]. Factors such as material composition, dimension, geometry, and design affect the longevity and risk of fracture of the prosthesis. The design of the connectors is one of the most relevant characteristics to assure the success of the all-ceramic FDP, considering that the fractures usually start from the gingival embrasure of this region and propagate until the occlusal surface [12,21-26]. Several studies [24,25,27-29] suggest that sharper connectors lead to higher stress concentrations at the gingival embrasure, which can lower the fracture resistance of three-unit FDPs. The distal connectors of posterior prostheses usually receive a higher load and, consequently, have a higher incidence of fractures when compared with the mesial connectors [12,21]. Furthermore, a previous FEA study [30] concluded that the stresses were concentrated at the distal connector in all models tested. In this study, the design of the distal connector of a three-unit FDP was modified to evaluate the stress distribution with varying radii of gingival curvature.

Traditionally, three- and four-point-bending in vitro testing methods have been broadly used to assess the fracture strength of all-ceramic FDPs. However, physical tests are (1) time-consuming and costly; (2) technique-sensitive; (3) unable to illustrate the stress distribution, and (4) subject to flaw variations in the specimen [10,12]. Finite element analysis (FEA) allows the calculation and observation of the stress distribution in each part of a geometrically complex structure such as FDP. With this method, we can analyze patient-based models obtained by image acquisition (3D scanners or computer tomography) or models created based on literature data. The modification of specific parameters like material properties and applied load provides accurate models that can be tested in a reliable, relatively fast, economically reasonable, and non-destructive way. Maximum principal stress represents the magnitude of tensile stress and is commonly used as a failure criterion for brittle materials such as all-ceramic FDPs [31].

FEA has also proven to be a powerful tool to test the resistance of materials and simulate distributions of stresses on novel products or clinically established protocols, with a potential application in cases of full-arch rehabilitation with zirconia [32,33], calvarial bone grafts [34], new types of internal connection between the abutment and implant [35], and the impact of occlusal contacts on temporomandibular disorder [36]. In addition, CAD/CAM systems and intraoral scanners have been used conjointly to speed productivity and improve the clinical workflow. Intraoral scanners substitute the traditional impressions, creating accurate digital models while CAD/CAM systems enable the development of new materials with better properties, aesthetics, and steady quality [37].

In the current study, the design of the distal connector of a retrieved three-unit all-ceramic FPD was modified. Models with three different radii of curvatures of the gingival embrasure (0.25 mm, 0.50 mm, and 0.75 mm) were created and analyzed by FEA. The purpose of this study was to validate clinical observations [38,39] and understand the importance of the radius of curvature of the gingival embrasure on the biomechanical behavior of fracture in implant-supported all-ceramic FDPs. The null hypothesis was that the distal connector with the broader curvature of gingival embrasure would present the lowest Maximum Principal Stresses on the gingival aspect of veneer and that framework layers and would promote more uniform stress distribution.

## 2. Materials and Methods

Two titanium dental implants (Astra Tech Osseospeed, Ø 4.0 and 4.8 mm, Dentsply Sirona, York, PA, USA), their suitable zirconia abutments, screws, and a patient-retrieved three-unit all-ceramic fixed dental prosthesis were scanned using a micro-CT scanner (Skyscan1172, Micro Photonics Inc.; Aartselaar, Belgium). The following parameters were used to conduct the scanning: (1) accelerating current of 100 μA and (2) a voltage of 100 kV. An exposure time of 1264 ms per frame was used to produce a TIFF (Tagged Image File Format) file from a Hamamatsu 10 MP camera. Sliced images from all physical components were generated at a resolution of 34.4 μm and reconstructed by NRecon software (Skyscan, Microphotonics Inc.; Aartselaar, Belgium).

The reconstructed tomographic image slices were imported to be processed into 3D models using interactive medical-image-processing software (Synopsys Simpleware; Mountain View, CA, USA). Individual masks of all components were produced by thresholding tools based on their greyscale values. Boolean operations, 3D editing, Recursive Gaussian filter, and Create object tools were used to correct artifacts, imperfections, and to generate 3D objects such as the simulated bone and fillings inside the abutments. Morphological close and Boolean tools were used on the abutments to produce 100 μm cement layers.

**Figure 1.**
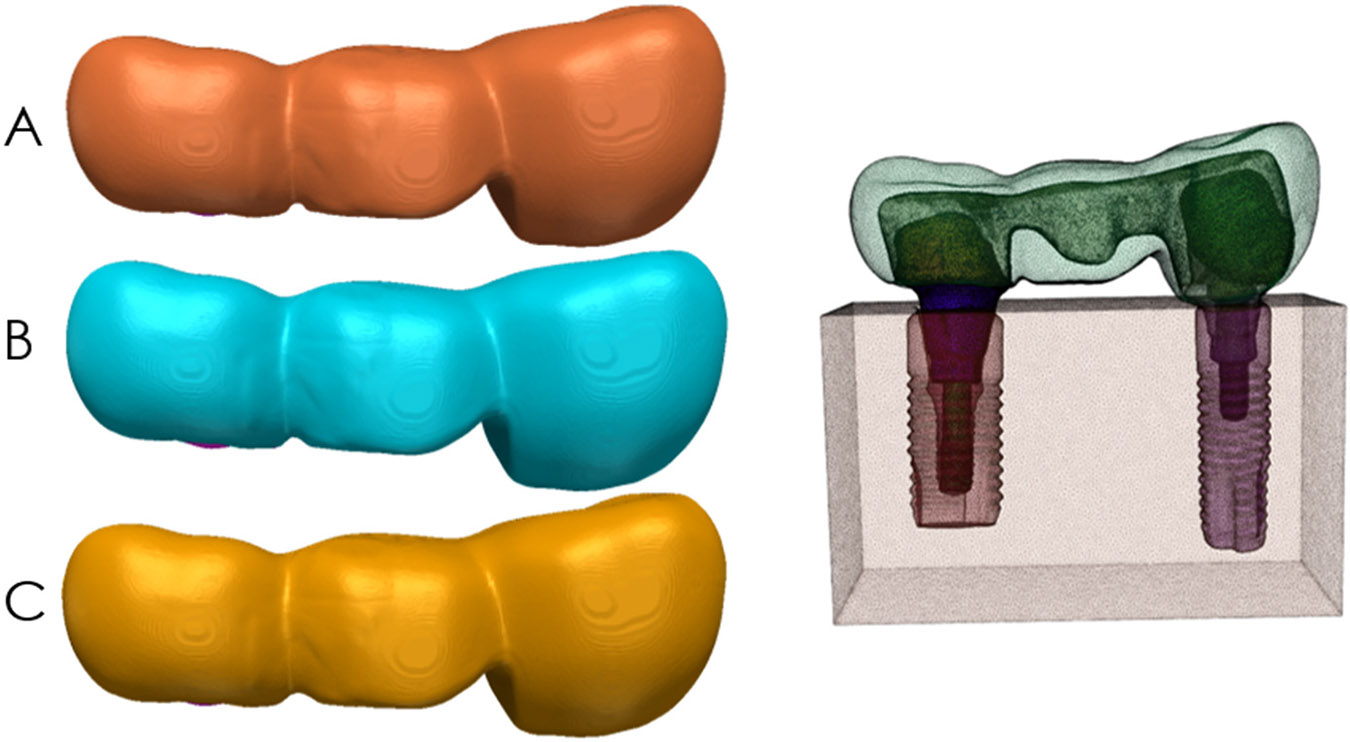
On the left: models (**A**–**C**) with 0.25 mm, 0.50 mm, and 0.75 mm radius of gingival embrasure respectively. On the right: Model B assembled.

The radius of curvature of the gingival embrasure at the distal connector was altered to produce three models: model A with 0.25 mm, model B with 0.50 mm, and model C with 0.75 mm, as illustrated in Figure 1. All the other features were kept constant. A 5 mm distal connector height with 7.60 mm depth was used on the three models. All masks were converted into surfaces by STL (Standard Tessellation Language) files to describe surface geometry and imported to a new file in Simpleware to be assembled in their anatomic positions.

**Figure 2.**
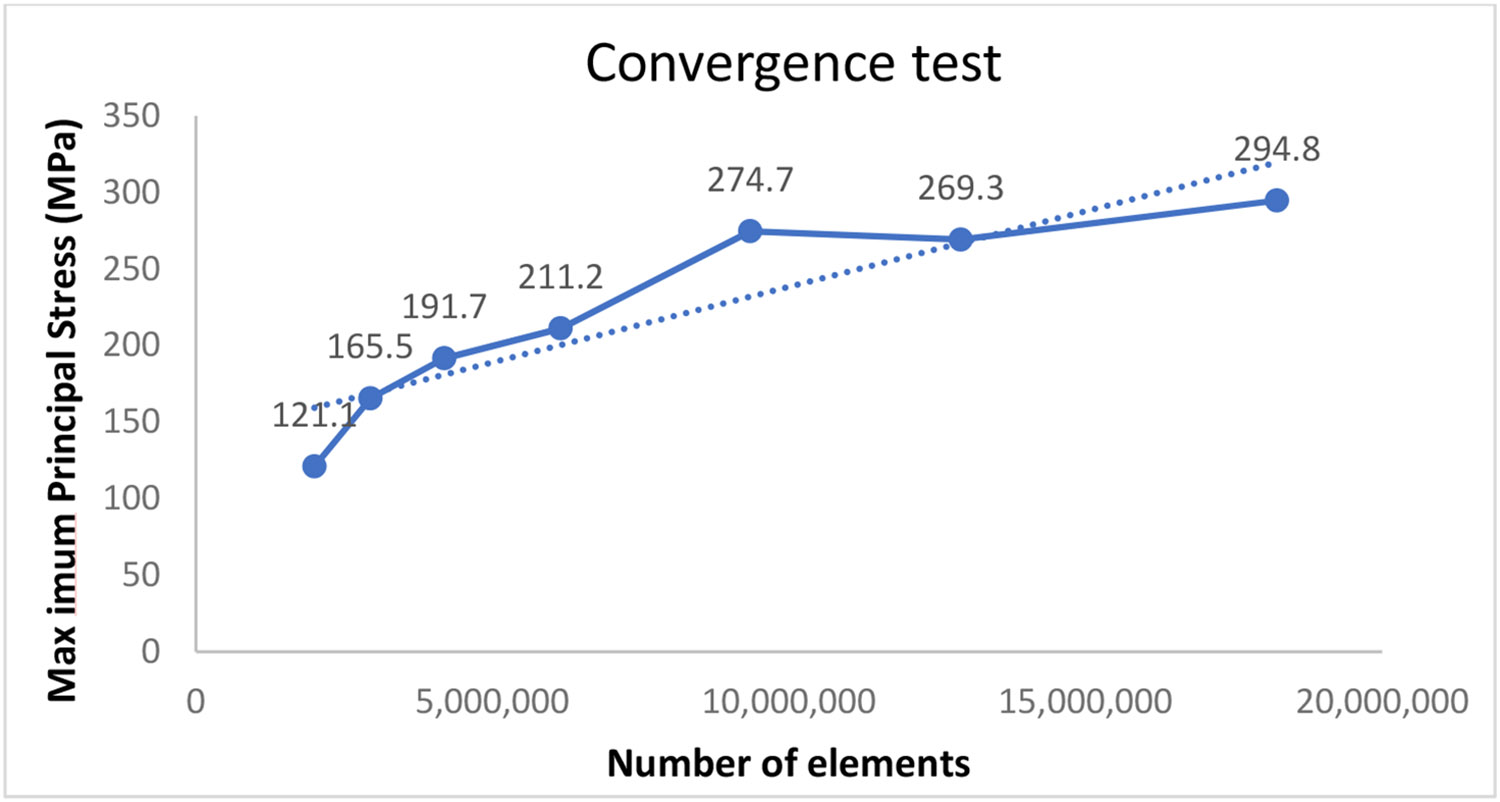
Convergence test data.

Seven mesh densities were used in an assembled model to conduct the convergence test. The INP files of the different mesh densities were imported and analyzed subsequently by a finite element analysis software (ABAQUS; Dassault Systèmes, Johnston, RI, USA). The number of elements for each mesh density and the Maximum Principal Stress on the corresponding volume model were recorded and plotted, as depicted in Figure 2. The −20 mesh density (274.7 MPa peak stress) was chosen as the most appropriate for further finite element analysis, demonstrating consistency of results independent of the mesh refinement and the lower computational effort. The number of tetrahedral elements was 9,317,088 for model A, 9,320,364 for model B, and 9,351,951 for model C when assembled and meshed (with −20 mesh density) for subsequent finite element analysis.

All materials were considered isotropic, homogeneous, and linear elastic. The material properties used for each component of the models are listed in Table 1 [40]:

Boundary condition was applied on all the external surface nodes of the bone, preventing displacement and rotation. A vertical load of 110 N was applied at the central fossa of the pontic. The axial compressive load was equally divided and applied onto 500 nodes (0.22 N/node). After a full analysis of the models, the Maximum Principal Stress data was collected. This stress was chosen because ceramics are more sensitive to tensile stress concentration due to their brittle nature. The distribution of stresses, peak stresses from the assembled mesh volumes and the ceramic veneer, framework, and abutment layers were recorded and analyzed.

## 3. Results

**Figure 3.**
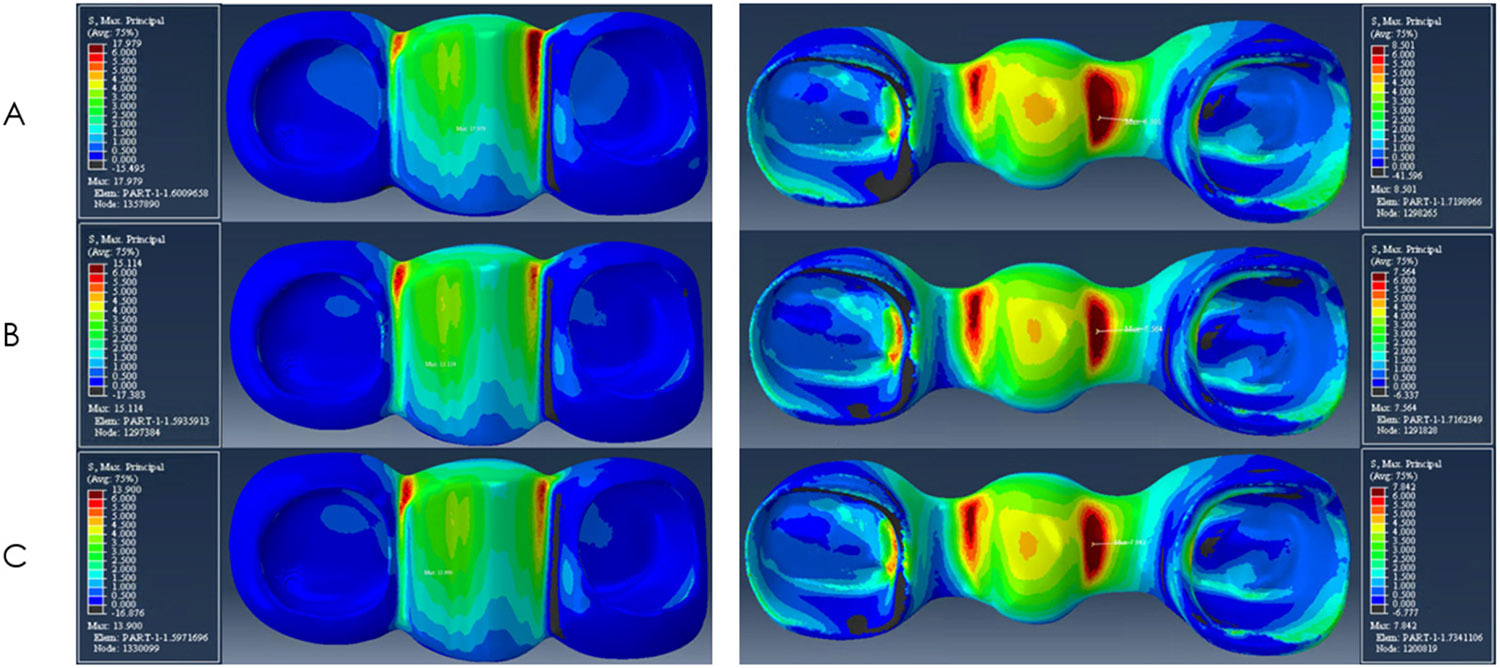
Stress distribution for models (**A**–**C**) on veneer (left column) and framework (right column).

The peak stresses in models A, B, and C were observed at the superiobuccal portion of the mesial implant. Model A, with a 0.25 mm radius of gingival embrasure presented the highest peak stress value (225 MPa) among all three models. The peak stresses in model B and model C were 189 MPa and 220 MPa, respectively. The distribution of Maximum Principal Stress within the ceramic veneer and zirconia framework is shown in Figure 3. The red colors represent the higher tensile stresses and the blue colors represent the lower tensile stresses. The peak stresses within the veneers were observed at the occlusal surface of the pontic, near the loading area for all models. The veneer peak stress values were 18 MPa for model A, 15 MPa for model B, and 14 MPa for model C. The peak stresses on the gingival embrasure of the veneer were observed at the mesial connector on model A (8 MPa), and at the distal connectors on model B (7 MPa) and C (6 MPa).

Within the framework, the peak stresses were located at the gingival aspect of the mesial connectors for all the models. The peak stress values on the framework were 8.5 MPa for model A, 7.5 MPa for model B, and 6.3 MPa for model C as depicted in Figure 3.

**Figure 4.**
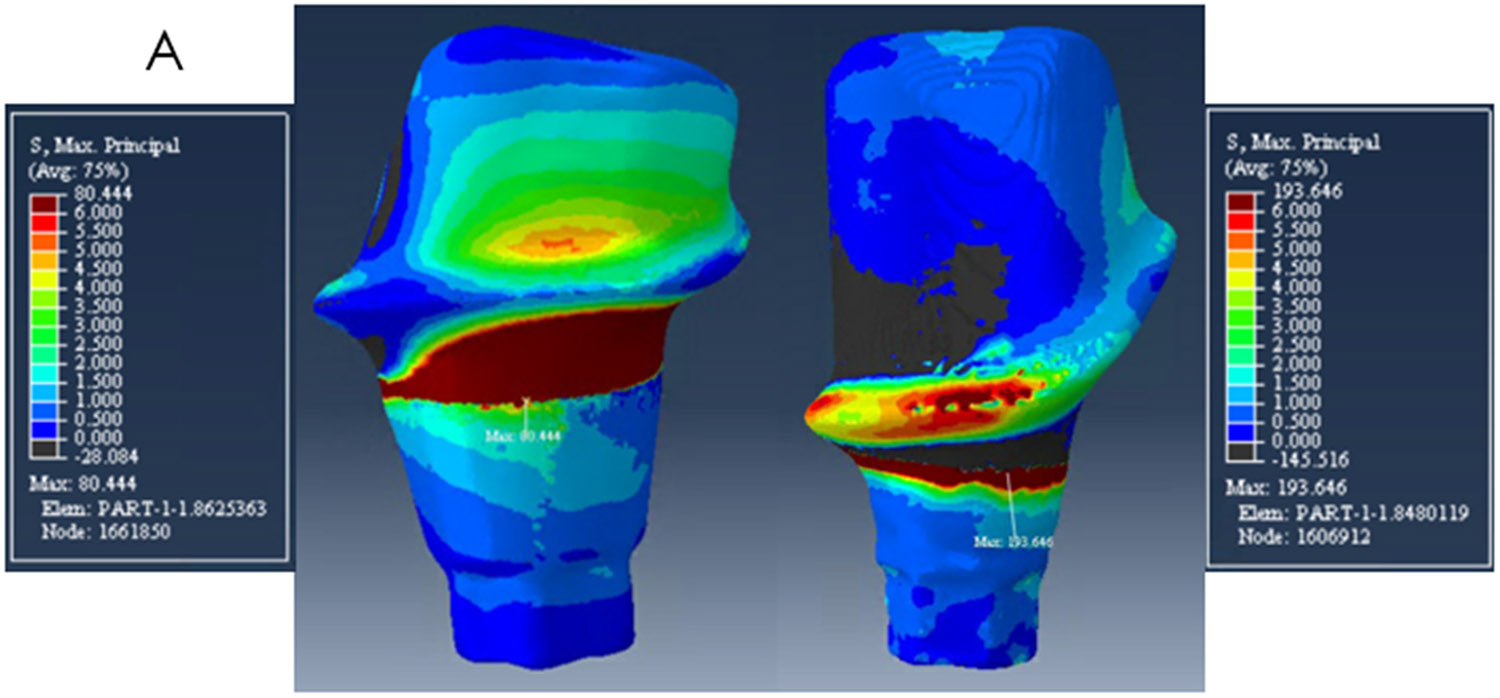
Distribution of Maximum Principal Stress on model A. The mesial abutment is on the right with a buccal view and the distal abutment on the left with a lingual view.

**Figure 5.**
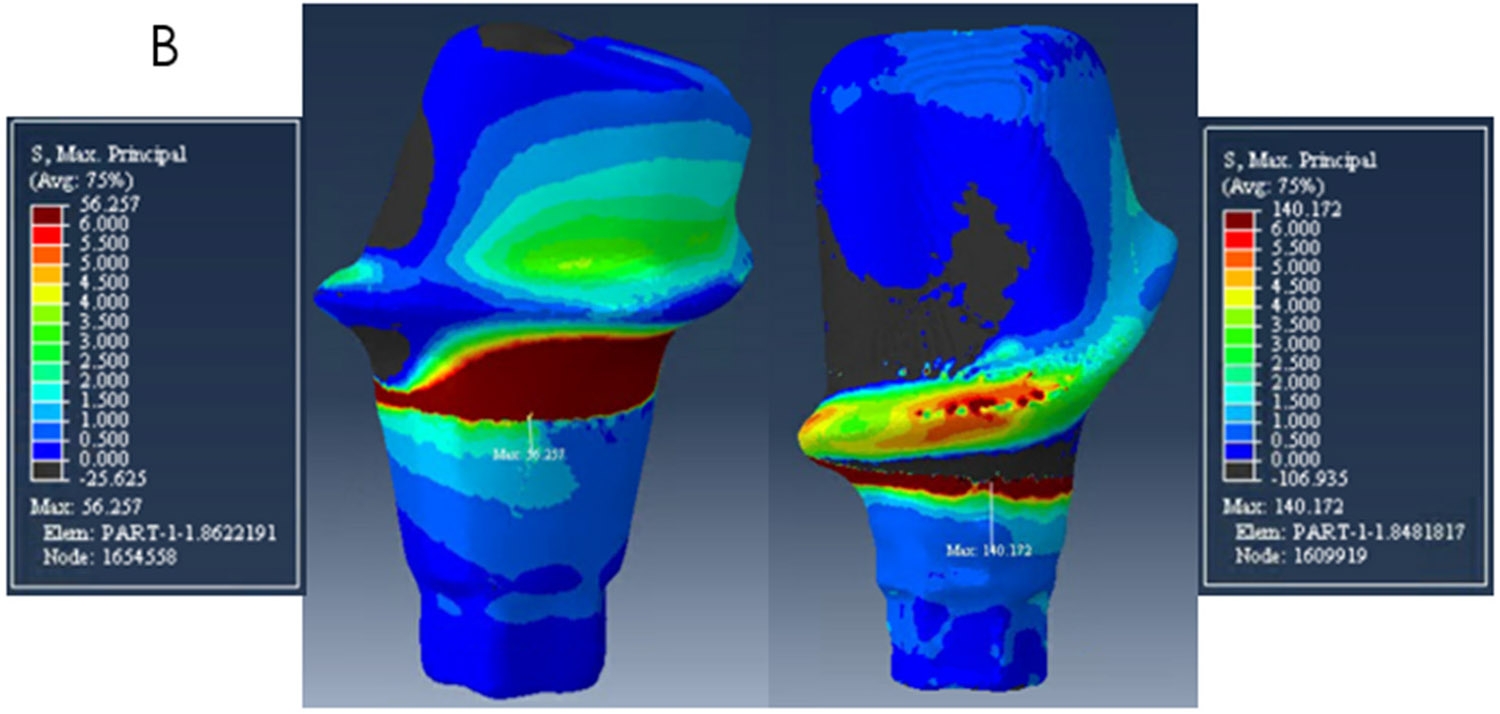
Distribution of Maximum Principal Stress on model B. The mesial abutment is on the right with a buccal view and the distal abutment on the left with a lingual view.

**Figure 6.**
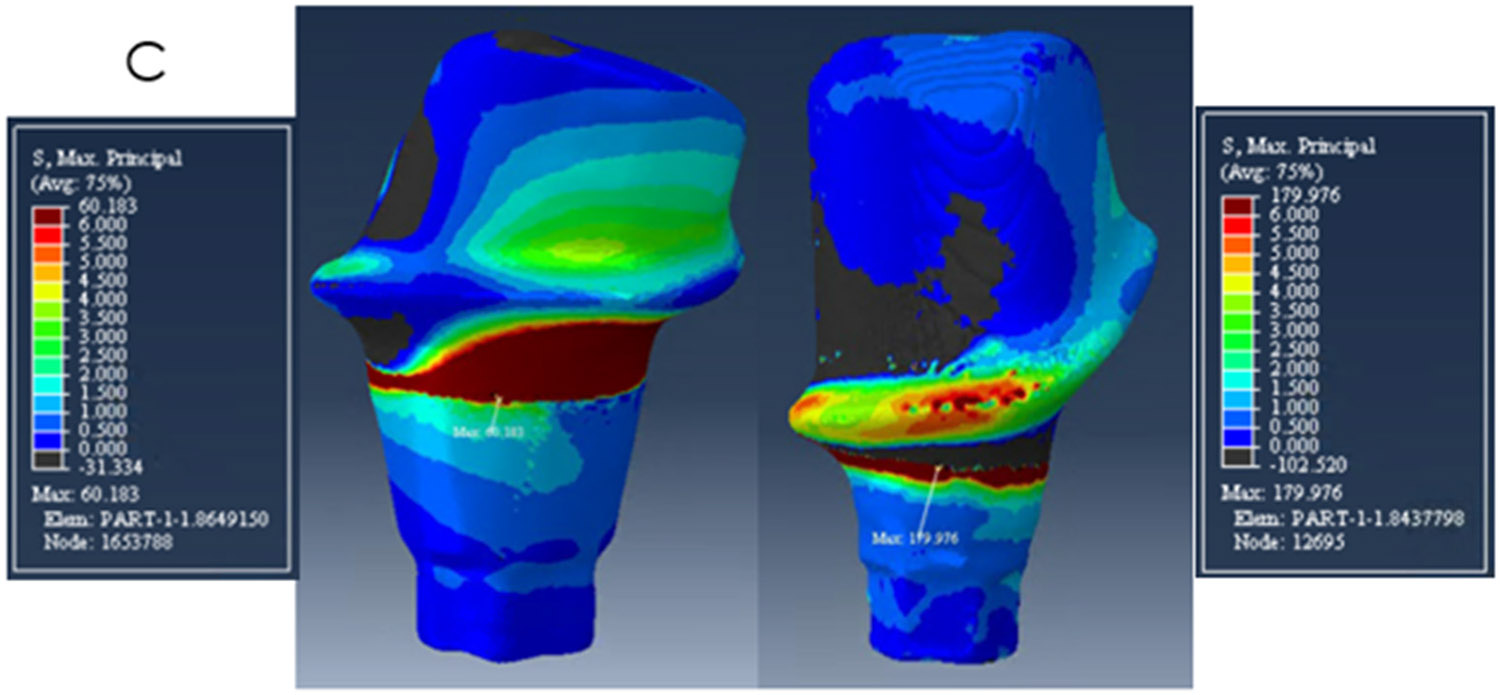
Distribution of Maximum Principal Stress on model C. The mesial abutment is on the right with a buccal view and the distal abutment on the left with a lingual view.

The zirconia abutments presented high-stress values at the neck area, at the buccal side on the mesial abutment, and on the lingual side on the distal abutment. The peak stresses were 194 MPa, 140 MPa, and 180 MPa on the mesial abutment and 80 MPa, 56 MPa, 60 MPa on the distal abutment for models A, B, and C respectively as shown in Table 2 and Figures 4-6.

## 4. Discussion

Several other studies [27,28] have used the FEA method to analyze the impact of the curvature of the gingival embrasure on the stress distribution and fracture resistance of all-ceramic three-unit posterior FDPs. The results were in agreement with the present study, wherein we observed that the sharper connector embrasure results in a higher concentration of tensile stress, likely leading to lower fracture resistance. One of the studies [27] compared the outcomes of models with 0.25 mm and 0.45 mm radii of gingival embrasure under a 100 N applied load at the central fossa of the pontic and concluded that the model with a smaller radius of curvature presented the higher tensile peak stresses (21 MPa) at the gingival embrasure. In another study [28], the same radii (0.25 mm and 0.45 mm) were used and the models were subjected to 400, 600, and 800 N vertical loads at the central fossa of the pontic. They concluded that the fracture resistance of posterior FDP under high loads can be increased by using greater radii of curvatures of the gingival embrasure.

Physical specimens tested regarding the influence of the connector design also corroborated the findings observed in this study. After testing forty-eight FDPs [29] with different connector designs, the results revealed that by increasing the radius of the gingival embrasure from 0.6 to 0.9 mm, a 20% increase in fracture strength was observed within a zirconia FPD with 3 × 3 mm connector dimension. Oh and Anusavice [24] tested 10 all-ceramic FDP specimens and concluded that, by increasing the radius of gingival curvature from 0.25 to 0.90 mm, the mean failure load was increased by 140%. Hamza et al. [41] evaluated the connector design using 20 rectangular zirconia bar-shaped specimens that simulated a three-unit FDP. The specimens designed with a broad radius of curvature (0.6 mm) presented higher flexural strength when submitted to a three-point bending test than the sharp ones (0.1 mm).

**Figure 7.**
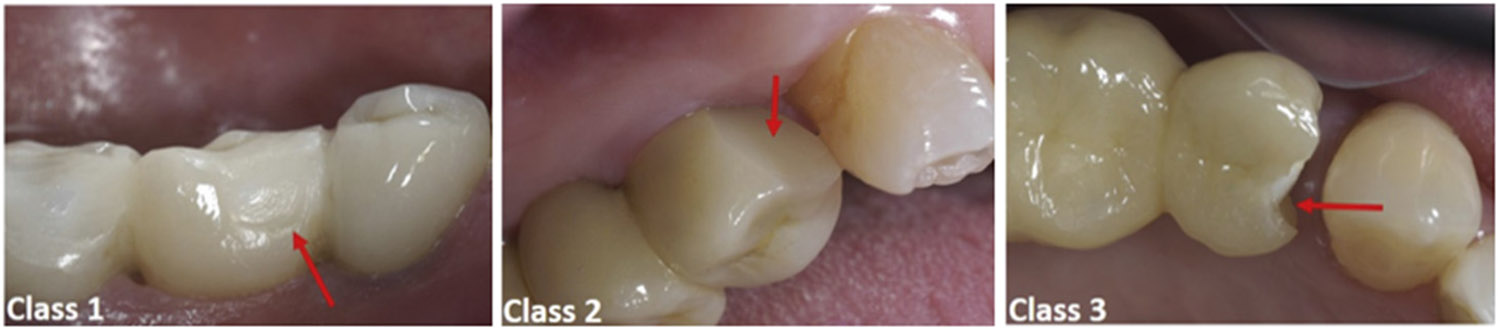
Clinical images of chipping fracture on all-ceramic FDPs, accordingly with their classification. Adapted with permission from Ref. [39]. Copyright 2021 Elsevier.

The results on models A, B, and C in this study demonstrated that the presence of peak tensile stresses near the loading area, independent of the size of the radius of curvature, could be responsible for chipping fracture, which is the most common cause of failure of multilayered FDPs [42-44]. It has been found that the stress values at the connector area are much lower than the flexural strength of the zirconia (800–1500 MPa) [10], indicating a low chance of failure in this area. This can be explained by the observations in two clinical follow-up studies [38,39], which analyzed a total of 88 all-ceramic implanted supported FDP with 0.25 mm, 0.50 mm, and 0.75 mm curvatures of gingival embrasure. No fractures occurred within the connector or embrasure areas. Chipping fractures occurred almost exclusively on the glass-ceramic veneers and were classified as: (1) Class 1—no intervention needed; (2) Class 2—repairs warranted and; (3) Class 3—remake of FDP required (Figure 7) [39]. In addition, no statistically significant correlation was found between the curvature of gingival embrasure and the survival probability of implant-supported FDPs with zirconia frameworks.

In this study, a clinical scenario was meant to be reproduced. Two implants with different diameters (Ø 4.0 and 4.8 mm) and their respective zirconia abutments were used in the models. The wider implant was placed at the posterior site to simulate anatomical restrictions with the necessity to endure higher loads. Both implants also presented slight angulations, a feature common in clinical scenarios. The patient-retrieved all-ceramic prosthesis was simplified but the main characteristics were maintained. The modification of only the posterior connector was based on a previous and related study [30] that demonstrated high tensile stress concentration on this site. The combination of all these features in addition to the modification of the curvature of gingival embrasure has influenced the distribution of the stresses on the components. It can be exemplified by the fact that the mesial and distal abutments of model B (which simulated the 0.50 mm radius of gingival embrasure) presented the lowest peak values for the set of parameters used in this study. This result demonstrates the fact that the distribution of the forces in an assembly prosthesis does not rely on only one parameter.

Many studies [11,12,24,25,27-30,38,39,42,45-51] have investigated an array of factors that could impact the survival rates and success of all-ceramic prostheses, including the radius of curvature of the gingival embrasure. Material properties of different ceramics [2,3,17], connector height and width [24,27,38,45,47], framework/veneer ratio [30], loading conditions [42], and patient factors [52] have been associated with a change in the lifetime of the restorations. Another factor to be considered is the crown/implant (C/I) ratio and its impact on the failure of implant components and cervical bone loss [51] under fatigue and various loads [53,54]. In this study, the peak stresses in models A, B, and C were observed at the superiobuccal portion of the mesial implant, which is longer and narrower than the posterior implant and has a higher crown/implant ratio. A recent FE study [55] showed that, with an increasing C/I ratio, the stress distribution in bone and implant increases. Such results are in agreement with the results of our study. However, in a systematic review [56], contradictory results were found: the highest C/I ratios presented less peri-implant marginal bone loss. Another systematic study [57] concluded that the increase in the C/I ratio does not seem to have a direct relationship with the increase in marginal bone loss and prosthetic failure. More research should be conducted regarding this topic as the results are not confluent and many factors can influence the outcomes.

This study demonstrated that FEA is an excellent tool to understand the distribution of the stresses on a three-unit all-ceramic implant-supported FDP with different radii of curvature of gingival embrasure. Therefore, in future studies, different loading conditions, material choices, and other geometric parameters can be simulated and tested. However, even showing reliable results and reducing costs compared with physical specimens testing, the 3D numerical simulations present limitations. In the current study, all the materials’ properties were assumed to be homogeneous, isotropic, and linear elastic, to simplify the models and facilitate better comparison between them. Additionally, in vivo conditions such as wet oral environment, intensity and frequency of the load, and distribution of masticatory forces were not considered.

## 5. Conclusions

Within the limitations of this study, the results demonstrated that the radius of curvature of gingival embrasure had a significant influence on the stress distribution of the components assessed. The assembly model with the smallest radius of curvature of gingival embrasure (0.25 mm) presented the highest Maximum Principal Stresses when compared with the models with a broader radius (0.50 mm and 0.75 mm). Furthermore, the peak tensile stresses at all adjacent structures were higher within the 0.25 mm model, while the 0.50 mm and 0.75 mm models presented similar values and better stress distribution. More attention should be paid to the neck area of the mesial abutment due to the high stress values observed among all the models analyzed.

## Figures and Tables

**Table 1.** Material properties of the components of the mesh volumes [40].

	Structures	Young’s Modulus (MPa)	Poisson’s Ratio
Porcelain	Veneer	70,000	0.19
Zirconia	Framework Abutments	210,000	0.30
Resin cement	Fillings Cement layer	8300	0.30
Titanium	Implants Screws	110,000	0.35
Bone	Cuboid (bone)	13,700	0.30

**Table 2.** Peak stress values for mesial and distal abutments on models A, B, and C.

	Model A	Model B	Model C
Mesial abutment	194 MPa	140 MPa	180 MPa
Distal abutment	80 MPa	56 MPa	60 MPa
